# Risk Factors of Postoperative Meningitis in Lateral Ventricular Trigone Meningiomas: A Clinical Analysis of 64 Patients

**DOI:** 10.3389/fsurg.2022.916053

**Published:** 2022-05-25

**Authors:** Xiaodi Han, Tianhao Hu, Run Wang, Longjie Li, Juanhan Yu, Li Zhang, Sheng Han

**Affiliations:** ^1^ Department of Neurosurgery, The First Hospital of China Medical University, Shenyang, China; ^2^ Department of Neurosurgery, Huazhong University of Science and Technology Union Shenzhen Hospital (Nanshan Hospital), Shenzhen, China; ^3^ Department of Pathology, First Affiliated Hospital and College of Basic Medical Sciences, China Medical University, Shenyang, China

**Keywords:** lateral ventricular trigone meningioma, postoperative meningitis, blood loss, risk factor, cerebral spinal fluid

## Abstract

**Purpose:**

Patients with intraventricular tumors are more susceptible to postoperative meningitis (POM) than other intracranial tumors. In this study, we explored the risk factors of POM in lateral ventricular trigone meningiomas (LVTMs).

**Methods:**

Clinical features of 64 patients with LVTMs were analyzed. Age, gender, body mass index, medical history, intraoperative blood loss (IBL), intraventricular drainage placement, surgical duration, tumor grade, postoperative tumor cavity hemorrhage, and tumor size were included in univariate and multivariate analyses of POM.

**Results:**

Of the 64 patients, 14 patients (21.9%) received diagnosis of POM. The univariate analysis revealed IBL ≥400 mL (odds ratio [OR], 9.012; *p *= 0.003), tumor size ≥50 cm^3^ (OR, 3.071; *p *= 0.080), and surgical duration ≥5 h (OR, 2.970; *p *= 0.085) were considered possible risk factors for POM (*p* < 0.10). Tumor size (*R* = 0.514) and surgical duration (*R* = 0.624) were significantly correlated with IBL (*p *< 0.05). In the multivariate analysis, only IBL was found to be an independent risk factor for POM.

**Conclusion:**

The IBL ≥400 mL is independently associated with the increased risk of POM in LVTM patients. Our results demonstrate the importance of controlling IBL for preventing POM, especially in large tumors and long surgeries.

## Introduction

Meningioma is the second most common intracranial tumor in adults, with an incidence rate of 1.5–5.5/100,000 (1–5). Whereas the lateral ventricular trigone meningioma (LVTM), which grows in the deep area of cerebral hemisphere, accounts for only 0.39%–2.3% of all meningiomas (6–9). However, LVTMs are often detected in giant tumors with hyper-vascularization due to their unique location (10). Additionally, LVTMs are surrounded by the cortical centers and fibers associated with speaking, making surgical treatment even more challenging.

Postoperative meningitis (POM) is a challenging problem for all neurosurgeons due to severe complications. Moreover, the complications lengthen the hospital stay and increase the medical costs. Additionally, it can also cause serious neurological dysfunction and even death. Unfortunately, POM is difficult to treat. According to previous reports, the incidence of POM ranges from 0.8%–7% for all craniotomies and 19.8% for intraventricular tumor surgeries (11, 12). Intraventricular meningiomas present a much higher incidence of POM than meningiomas located in other areas (11–13). However, risk factors for POM remain unclear for LVTM surgeries. Therefore, in the current study, we explored the risk factors of POM in LVTM patients (*n* = 64) to bring insight for reducing the incidence of POM.

## Materials and Methods

### Patients

We reviewed a total of 2,573 consecutive patients with the diagnosis of intracranial meningioma in the First Hospital of China Medical University from December 2010 to May 2018. Intraventricular meningiomas accounted for 2.8% (71/2,573) of all meningiomas.

Exclusion Criteria: Patients with meningiomas in the third or fourth ventricle, lacking preoperative imaging data, and who had not received an operation in the First Hospital of China Medical University were excluded.

Finally, 64 (2.5%) patients with LVTMs were included for the analysis. All of the patients underwent routine preoperative physical, radiological, and laboratory examinations. The study protocol was approved by the institutional review board of the First Hospital of China Medical University. In addition, written informed consent was obtained from each patient to use clinical data for future research.

**Figure 1:**
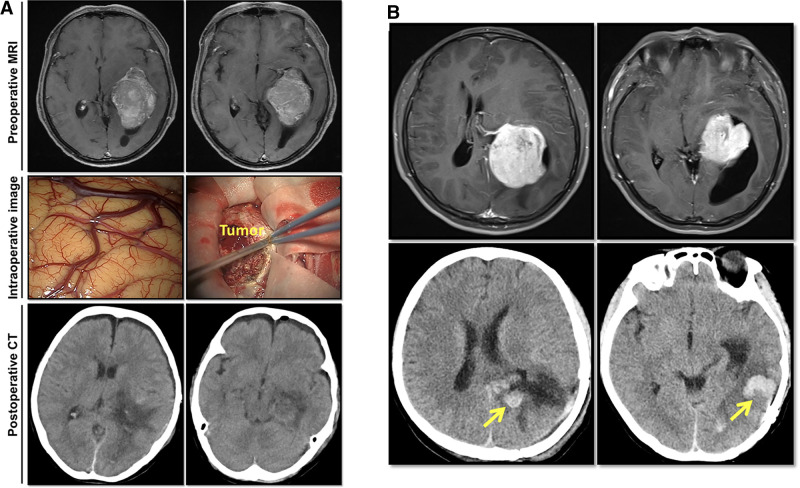
Imaging studies of two representative lateral ventricular trigone meningioma (LVTM) patients. (**A**) A 63-year-old female patient presented with dizziness and headache. Preoperative axial enhanced T1-weighted magnetic resonance imaging (MRI) showed a huge mass in the left trigone area. Intraoperative observation and postoperative computed tomography (CT) imaging showed a total tumor resection. On a 6th postoperative day, she had a fever of 38.5°C with a headache and meningeal signs. The CSF test showed elevated white cell count (394 10^6^/L), protein level (>3,000 mg/L), and decreased glucose level (<1.1 mmol/L). Lumbar drainage was performed, and meropenem was used according to empirical therapy. Due to the negative CSF smear and culture results, meropenem was used continuously. On the 20th postoperative day, the patient had maintained a normal temperature for a week. CSF tests were repeated three times with normal results. The drainage was removed, and the meropenem was stopped. The patient was discharged from the hospital on the following day. (**B**) A 35-year-old male patient presented with a headache. Preoperative axial enhanced T1-weighted MRI revealed a large mass in the left trigone area (upper image). Postoperative 24-h CT showed total tumor resection with the tumor cavity hemorrhage (lower image). The patient showed mild headache without fever disturbance of consciousness. On a postoperative day 7, he had a continuous fever of 39°C. The CSF test showed elevated white cell count (754 10^6^/L), protein level (>3,000 mg/L), and decreased glucose level (<1.1 mmol/L). Lumbar drainage was placed, and meropenem was used according to empirical therapy. As the CSF culture showed methicillin-resistant *Staphylococcus aureus*, meropenem was replaced by vancomycin. On the 33rd postoperative day, the patient had maintained normal temperature for a week with 3 times of normal CSF tests. The drainage was removed, and vancomycin was stopped. The patient was discharged from the hospital on the following day.

All patients received 1.0–2.0 g of intravenous ceftriaxone intraoperatively for infection prophylaxis as described previously (14–16). A second dose of ceftriaxone was administered in patients whose operation had lasted more than 3 h (16). A trans-sulcal or transcortical temporal-occipital approach was adopted, depending on the size and location of the tumor. Gross total resection was performed in all the patients (Figure 1A). In 13 (20.3%) patients, drainage was left in the ventricular tumor cavity to drain the residual debris and blood after tumor removal (17). The drainage was removed no more than five days after surgery. Pathological diagnoses were reported by the Pathology Department of China Medical University. All of the surgical procedures were performed by neurosurgeons with more than 10 years of experience.

### Postoperative Meningitis (POM)

POM was determined as previously described (18). Briefly, patients with POM presented with the following symptoms and signs: fever >38°C, headache, meningeal signs. In addition, each patient had at least one of the following laboratory results: (1) organisms cultured from the cerebral spinal fluid (CSF); (2) elevated white cell count (0–8 10^6^/L), protein level (120–600 mg/L), and decreased glucose level (2.2–3.9 mmol/L) in the CSF; (3) organisms observed in the CSF smear examination; and (4) positive antigen test in the CSF.

### Data Collection

The clinical data were obtained from our hospital electronic information system. Patient information, including age (<50 vs. ≥50 years), gender, body mass index (BMI), past medical history (hypertension and diabetes), intraoperative blood loss (IBL), intraventricular drainage placement, surgical duration (<5 h vs. ≥5 h), tumor grade, postoperative tumor cavity hemorrhage, and tumor size (<50 cm^3^ vs. ≥50 cm^3^) were included in univariate and multivariate analyses of POM.

### Statistical Analyses

Statistical analyses were performed using SPSS version 26.0 (IBM Corporation). The chi-squared test was conducted, and factors with *p* < 0.10 were used to input into multivariate logistic regression analysis. *p *< 0.05 was considered statistically significant.

The study population was divided into two groups for validation analyses depending on the patient’s admission time. The first 32 patients from December 2010 to January 2015 were included in group A, while the last 32 patients, from March 2015 to May 2018, were considered group B.

## Results

### Clinical Features of the LVTM Patients

As shown in Table 1, among the 64 cases, there were 24 men (37.5%) and 40 women (62.5%). The average age was 47.39 ± 13.55 years (16–72 years). According to the nearest integers of average values, the cutoff values for age, BMI, tumor size, surgical duration, and volume of IBL were determined as 50 years, 24 kg/m^2^, 50 cm^3^, 5 h, and 400 mL, respectively. There were 31 patients (48.4%) with age ≥50 years and 33 patients (51.6%) with age <50 years. The average tumor size was 52.78 ± 65.09 cm^3^ (1.9–301.4 cm^3^). There were 36 patients (56.2%) with tumors <50 cm^3^, while 28 patients (43.8%) with tumors ≥50 cm^3^. The surgery lasted for an average of 4.68 ± 1.88 h (2–11 h), whereas the average volume of IBL was 359 ± 342 mL (range, 50–1,500 mL). In 18 patients (28.1%) surgery lasted ≥5 h, while in 46 patients (71.9%) it lasted <5 h. There were 52 patients (81.2%) with IBL <400 mL and 12 patients (18.8%) with IBL ≥400 mL. There were 13 patients (20.3%) who had hypertension and no patients with diabetes. Intraventricular drainage was placed in 13 patients (20.3%). Eight patients had postoperative tumor cavity hemorrhage (12.5%; Figure 1B), and two (3.1%) of the patients required secondary surgery to remove the hematoma. Two patients (3.1%) recived a ventriculoperitoneal shunt within one year after the operation. Among the 64 patients tumors, 56 (87.5%) belonged to grade I; seven (10.9%) belonged to grade II, and one (1.6%) belonged to grade III (Table 1) (19). There was no cystic meningioma in the series of cases. Among 14 patients, one patient’s CSF smear showed Gram-positive bacteria (7.1%), and another patient’s CSF culture showed Staphylococcus aureus (7.1%).

**Table 1:** The clinical characteristics of the 64 patients with LVTMs.

Category	No. (%) or Mean ± SD [range]
Gender	
Female	40 (62.5)
Male	24 (37.5)
Age (years)	
Mean ± SD	47.39 ± 13.55 [16–72]
<50	33 (51.6)
≥50	31 (48.4)
BMI (kg/m^2)^	
Mean ± SD	24.11 ± 3.88[16.0–35.2]
<24	31(48.4)
≥24	33(51.6)
Size of tumor (cm^3)^	
Mean ± SD	52.78 ± 65.09 [1.9–301.4]
<50 cm^3^	36 (56.2)
≥50 cm^3^	28 (43.8)
Volume of blood loss intraoperative (mL)	
Mean ± SD	359.06 ± 342.03 [50–1,500]
<400 mL	52 (81.2)
≥400 mL	12 (18.8)
Surgical duration (h)	
Mean ± SD	4.68 ± 1.88 [2.0–11.0]
<5 h	46 (71.9)
≥5 h	18 (28.1)
Grades of tumor	
I	56 (87.5)
II	7 (10.9)
III	1 (1.6)
Hypertension	13 (20.3)
Intraventricular drainage	13 (20.3)
Tumor cavity hemorrhage	8 (12.5)
Postoperative meningitis	14 (21.9)

### Risk Factors for POM

**Figure 2:**
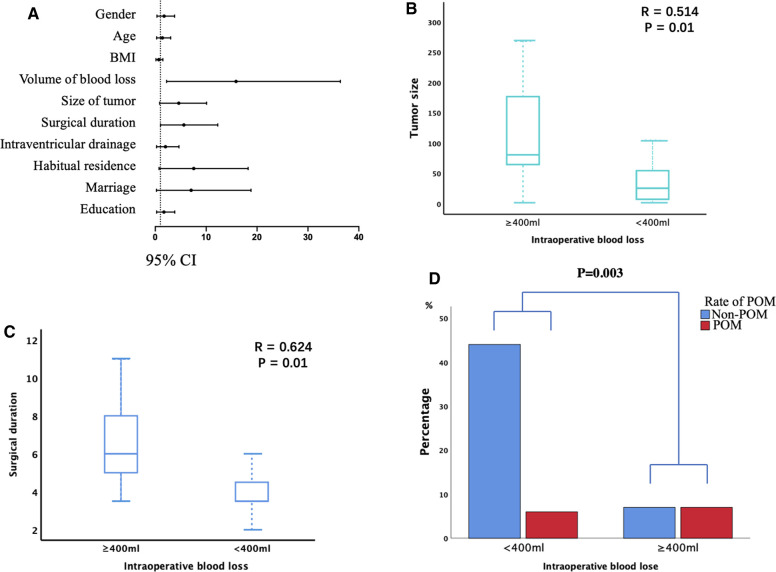
The risk factors for postoperative intracranial infection (POM). (**A**) Univariate analyses of the risk factors for POM. Odds ratios are shown with 95% CIs. (**B,C**) Tumor size (**B**) and surgical duration (**C**) were significantly associated with intraoperative blood loss (IBL). (**D**) IBL ≥400 mL was associated with a significantly higher incidence of POM.

The potential risk factors of POM are presented in Table 2. The univariate analysis revealed IBL ≥400 mL, tumor size ≥50 cm^3^, and surgical duration ≥5 h were considered possible risk factors for POM (*p* < 0.10; Figure 2A). These were included in the multivariate logistic regression analysis. According to the Pearson correlation analysis, tumor size (*R* = 0.514; Figure 2B) and surgical duration (*R* = 0.624; Figure 2C) were significantly correlated with IBL (*p *< 0.05). However, gender, age, BMI, and history of hypertension did not correlate with POM. Intraventricular drainage placement was not significantly associated with POM in this study.

**Table 2:** The results of univariate analysis.

Variable (%)	Assigned	Meningitis (*n* = 14)	Non-meningitis (*n* = 50)	*χ* ^2^	*p*	OR	95% CI
Gender	Female	9 (22.5)	31 (77.5)	0.024	0.876	1.103	0.321–3.787
	Male	5 (20.8)	19 (79.2)				
Age	<50	7 (21.2)	26 (78.8)	0.018	0.895	0.923	0.282–3.021
	≥50	7 (22.6)	24 (77.4)				
BMI	<24	9 (29.0)	22 (71.0)	1.802	0.179	0.437	0.128–1.490
	≥24	5 (15.2)	28 (84.8)				
Volume of blood loss	<400	7 (13.5)	45 (86.5)	9.012	*0.003*	9.000	2.226–36.382
	≥400	7 (58.3)	5 (41.7)				
Size of tumor	<50	5 (13.9)	31 (86.1)	3.071	*0*.*080*	2.937	0.856–10.082
	≥50	9 (32.1)	19 (67.9)				
Surgical duration	<5	7 (15.2)	39 (84.8)	2.970	*0*.*085*	3.545	1.023–12.290
	≥5	7 (38.9)	11 (61.1)				
Intraventricular drainage	Yes	3 (23.1)	10 (76.9)	0.000	1.000	1.091	0.255–4.663
	No	11 (21.6)	40 (78.4)				

Furthermore, IBL, tumor size, and surgical duration were analyzed through multivariate analysis. As shown in Table 3, only IBL ≥400 mL was an independent risk factor for POM (*p* = 0.018; Figure 2D). Our results indicated that larger tumor size and longer surgical duration might cause more IBL, further increasing the risk of POM. Finally, a similar result was obtained in the validation analysis of groups A and B: only IBL ≥400 mL was an independent risk factor for POM (*P* = 0.049, 0.046 for groups A and B, respectively).

**Table 3:** The results of multivariate analyses.

	B	Standard deviation	Wald	df	*p*	Exp(B)	95% CI
Volume of blood loss	1.869	0.793	5.559	1	*0.018*	6.481	1.371– 30.650
Surgical duration	0.420	0.778	0.291	1	0.589	1.522	0.331–6.998
Size of tumor	0.689	0.702	0.996	1	0.326	1.993	0.504–7.883

### Treatment of POM

Patients with POM were treated with lumbar drainage and antibiotics (20). First, lumbar drainage was placed, and meropenem, vancomycin, or both were used as empirical therapy (21, 22). The next course of treatment depended on the results of the CSF tests. Among the 14 patients, nine patients (64.3%) received meropenem only; one (7.1%) received vancomycin only; and four patients (28.6%) received meropenem and vancomycin. The patients were cured within 8–39 days (18.57 ± 8.76 days). However, POM cases average length of postoperative hospital stay was 10 days longer than non-POM cases (*p *= 0.033).

## Discussion

POM is a common postoperative complication of neurosurgery, causing severe consequences (23–25). Previous studies have reported that patients with intraventricular tumors were more susceptible to POM than other intracranial tumors (12, 26, 27). Therefore, identifying the risk factors for POM in LVTM patients carries significant clinical value.

In this study, 64 LVTM patients underwent craniotomy, out of which 14 patients (21.8%) had the diagnosis of POM. Previously, Wang et al. reported a 19.8% incidence of POM, 1.7% incidence of postoperative hematoma evacuation, and 3.3% incidence of postoperative ventriculoperitoneal shunt in out of 121 LVTMs (12). Though they did not analyze the risk factors for POM, their results showed a close relationship between ventricular drainage and postoperative entrapped temporal horn. However, in our study, ventricular drainage was not identified as a risk factor for POM. Besides, ventricular drainage was only used in 20.3% of the patients, which was lower than the report of Wang et al. (87.6%). Nevertheless, the incidence of postoperative complications including meningitis, severe hemorrhage in the operative cavity and hydrocephalus requiring shunting in our series of cases was not significantly different from that in Wang’s series of cases. In addition, previous studies have reported that ventricular drainage increases the risk of POM (28–31). Thus, ventricular drainage should be used with caution in LVTM surgeries to prevent complications. Intraoperative ultrasound (IoUS) provides great help in neurosurgery, which can increase the chances of total resection and reduce the risk of postoperative bleeding in the surgical cavity. Neurosurgeons can get the real-time information about the anatomical relationship between tumor and its surrounding tissue (32). Therefore, tumors can be removed more precisely with reduced intraoperative blood loss and potentially decreased risk of postoperative meningitis.

We found that IBL ≥400 mL was an independent risk factor for the POM. This observation was in-concurrence with previous studies reporting the correlation between IBL and POM (33, 34). The large amount of IBL may compromise the patient’s immune system and adversely influence the physiology of local brain tissue. The coagulation pathway might also get activated, leading to the recruitment of inflammatory cells and cytokines. As a result, tissue hypoperfusion and ischemia-reperfusion injury might occur. Further, inflammatory reaction and an ischemia-reperfusion injury increase the vascular permeability damage the integrity of the endothelium, and blood-brain barrier, enhancing the risk of POM (35–38). Massive IBL may decrease the concentration of prophylactic antibiotics in the blood and reduce its preventive effects against infection (39–44). Moreover, massive IBL is associated with allogeneic transfusion. However, the transfusion of red blood cells can contribute to POM by enhancing the inflammatory reaction and suppressing immunity (45–47).

In addition, our results also indicate that large tumor size and long surgical duration were associated with increased IBL. This association further affects the incidence of POM. Large LVTMs are usually hypervascular and fibrous and require long-lasting operations. Moreover, cystic meningioma is a special kind of meningiomas with a relatively low occurrence of about 3.5% (48). There was no cystic meningioma in our series of cases. However, according to previous study, cystic meningiomas are known to increase the length of surgery and intraoperative blood loss (48). Thus, for cystic meningiomas, surgeons should pay more attention to the risk of postoperative meningitis.

Our results emphasized the importance of controlling intraoperative bleeding. Of note, preoperative embolization, meticulous intraoperative hemostasis, and early tumor devascularization may reduce the IBL and, in turn, decrease the risk of POM (32). However, when a massive IBL occurs, an additional dose of intraoperative antibiotics might be helpful to prevent POM. Importantly, POM significantly prolongs hospital stays, increases medical costs, and affects clinical outcomes. Thus, the prevention of POM may provide significant benefits to patients.

The current study had some limitations. One major limitation was the lack of distinction between bacterial and aseptic meningitis. As a result, they show similar symptoms (new headache, fever, seizures, etc.), CSF test (abnormalities of CSF cell count, glucose, and protein), except CSF culture and smear. However, CSF culture and smear show a low positive rate, which can be reduced by prophylactic antibiotics (49). In this study, all 14 patients with POM showed some improvement and finally recovered after antibiotic therapy, suggesting the potentially beneficial effect of antibiotic therapy.

## Conclusion

The IBL ≥400 mL is independently associated with the increased risk of POM in LVTM patients. Our results demonstrate the importance of controlling IBL for preventing POM, especially in large tumors and long surgeries.

## Data Availability

The raw data supporting the conclusions of this article will be made available by the authors, without undue reservation.
